# Recovery of Recombinant Crimean Congo Hemorrhagic Fever Virus Reveals a Function for Non-structural Glycoproteins Cleavage by Furin

**DOI:** 10.1371/journal.ppat.1004879

**Published:** 2015-05-01

**Authors:** Éric Bergeron, Marko Zivcec, Ayan K. Chakrabarti, Stuart T. Nichol, César G. Albariño, Christina F. Spiropoulou

**Affiliations:** Viral Special Pathogens Branch, Division of High Consequence Pathogens and Pathology, National Center for Emerging and Zoonotic Infectious Diseases, Centers for Disease Control and Prevention, Atlanta, Georgia, United States of America; Icahn School of Medicine at Mount Sinai, UNITED STATES

## Abstract

Crimean Congo hemorrhagic fever virus (CCHFV) is a negative-strand RNA virus of the family *Bunyaviridae* (genus: *Nairovirus*). In humans, CCHFV causes fever, hemorrhage, severe thrombocytopenia, and high fatality. A major impediment in precisely determining the basis of CCHFV’s high pathogenicity has been the lack of methodology to produce recombinant CCHFV. We developed a reverse genetics system based on transfecting plasmids into BSR-T7/5 and Huh7 cells. In our system, bacteriophage T7 RNA polymerase produced complementary RNA copies of the viral S, M, and L segments that were encapsidated with the support, in *trans*, of CCHFV nucleoprotein and L polymerase. The system was optimized to systematically recover high yields of infectious CCHFV. Additionally, we tested the ability of the system to produce specifically designed CCHFV mutants. The M segment encodes a polyprotein that is processed by host proprotein convertases (PCs), including the site-1 protease (S1P) and furin-like PCs. S1P and furin cleavages are necessary for producing the non-structural glycoprotein GP38, while S1P cleavage yields structural Gn. We studied the role of furin cleavage by rescuing a recombinant CCHFV encoding a virus glycoprotein precursor lacking a functional furin cleavage motif (RSKR mutated to ASKA). The ASKA mutation blocked glycoprotein precursor’s maturation to GP38, and Gn precursor’s maturation to Gn was slightly diminished. Furin cleavage was not essential for replication, as blocking furin cleavage resulted only in transient reduction of CCHFV titers, suggesting that either GP38 and/or decreased Gn maturation accounted for the reduced virion production. Our data demonstrate that nairoviruses can be produced by reverse genetics, and the utility of our system uncovered a function for furin cleavage. This viral rescue system could be further used to study the CCHFV replication cycle and facilitate the development of efficacious vaccines to counter this biological and public health threat.

## Author Summary

Crimean Congo hemorrhagic fever (CCHF) is a severe viral disease characterized by rapid-onset fever, hemorrhage, and high case fatality rates. CCHF virus (CCHFV), the causative agent of CCHF, is a negative-strand RNA virus of the family *Bunyaviridae* (genus *Nairovirus*). No specific treatments or efficacious vaccines exist to combat CCHF. To investigate molecular determinants of nairovirus pathogenesis and biology, we developed a reverse genetics system capable of generating CCHFV variants with genome sequences defined by the plasmids transfected into cells for virus recovery. Our system is the first to demonstrate that a nairovirus can be efficiently recovered from the simple transfection of plasmid DNA, paving the way for specifically editing genomes of CCHFV and other nairoviruses. Using this system, we engineered mutations blocking the cleavage of CCHFV’s non-structural glycoproteins at a motif recognized by the host protease furin. Using this furin-resistant CCHFV variant, we demonstrate that direct cleavage of the viral glycoprotein by furin results in a lag in virion production, revealing a function of these glycoproteins in efficient CCHFV replication. Our experiments highlight the utility of a reverse genetics system for developing viral variants for investigating CCHFV protein function and for rationally designing vaccine strains.

## Introduction

Crimean Congo hemorrhagic fever virus (CCHFV) is a severe human pathogen transmitted through tick bites or contact with infected animals or patients. In ~5–40% of the cases, patients suffer from profound hemorrhage leading to shock and death. CCHFV was first isolated in the Democratic Republic of the Congo in 1956, and later classified into the family *Bunyaviridae*, genus *Nairovirus* [1]. The family also includes the genera *Orthobunyavirus*, *Hantavirus*, *Phlebovirus*, and *Tospovirus*. Today, CCHFV is recognized as endemic in several countries of Africa, Europe, Asia, and the Middle East, where the main vector, hard ticks of the genus *Hyalomma*, are found [1]. Recent emergence and re-emergence of Crimean Congo hemorrhagic fever (CCHF) in Turkey, southern Russia, Balkans, Democratic Republic of the Congo, Sudan, Uganda, Pakistan, and India have spurred concerns over the spread of this disease to other countries where ticks vectors are present [2,3].

The initial phase of CCHF consists of an acute febrile prodrome indistinguishable from that caused by several other viral infections. However, in less than a week, CCHF often progresses into a life-threating illness characterized by leucopenia, high levels of pro-inflammatory cytokines, coagulopathy, liver necrosis, and hemorrhage [4]. These pathological findings likely reflect a pro-inflammatory immune response mounted against viral infection, and the resulting tissue damage. The high virulence, absence of effective treatments, and documented person-to-person transmission of CCHFV have justified its inclusion on the list of agents requiring biosafety level 4 (BSL-4), the highest level of containment, to perform experimental work [5].

The genome of nairoviruses is distributed on 3 negative-sense RNA segments designated small (S), medium (M), and large (L). The S segment encodes the nucleoprotein (N) that encapsidates the viral genome to form ribonucleoprotein particles (RNPs). The M segment encodes a ~1700 amino acid precursor that is processed into structural glycoproteins (Gn and Gc) that decorate the surface of virions, non-structural M protein (NSm) [6], and secreted non-structural glycoproteins (NSGs) GP85, GP160, and GP38 [7,8]. L segment mRNA is translated into an unusually large (~450 kDa) RNA-dependent RNA polymerase (L-RdRp) that replicates and transcribes viral RNA [9].

Nairovirus glycoprotein biosynthesis is more complex than that of other bunyaviruses in terms of size, functional domains, and the number of mature proteins produced [7,8]. Synthesis of the CCHFV M segment polyprotein is targeted to the endoplasmic reticulum, where the signal peptidase complex rapidly cleaves it into NSm, the membrane bound Gn precursor (PreGn), and the Gc precursor (PreGc) [7]. PreGn and PreGc are further modified by addition of N-linked sugars, and cleaved into Gn and Gc by highly specific host endoproteases in an early compartment of the secretory pathway. PreGn is processed by the site-1 protease (S1P, also known as subtilisin kexin isozyme-1 [SKI-1]), a member of the proprotein convertase (PC) family. PreGc is matured by an unidentified host convertase [10]. S1P activity leads to the selective incorporation of Gn and Gc into nascent virions [11], and to the secretion of NSGs containing 2 domains specific to nairoviruses: a heavily O-glycosylated and variable mucin-like domain (MLD), and a GP38 domain [8]. Three NSGs are observed in the extracellular milieu of CCHFV-infected cells: GP160 and GP85, which contain both MLD and GP38 domains; and GP38, a cleavage product of GP160/GP85 lacking the MLD domain [8]. It is currently unclear what accounts for the differential migration of GP160 and GP85 on SDS-PAGE, as neither tertiary structures nor glycosylation appear to contribute. GP85 probably contains a shorter polypeptide chain while still sharing common epitopes with GP160 in both MLD and GP38 domains [8,12]. The last processing step leading to GP38 secretion occurs at a canonical furin-like PC motif (RSKR↓) in a late compartment of the secretory pathway [8], most likely in the trans-Golgi network (TGN) where furin concentrates [13]. Cleavage of NSGs by furin and/or PCs with related specificity leads to the secretion of GP38.

Recovery of recombinant viruses from cloned complementary DNA allows the design of viruses with precisely defined genomic sequences. This permits researchers to elucidate viral protein function in the context of infection. Viruses with tailored genomes are useful for designing viral variants and live attenuated vaccines harboring engineered mutations and/or gene deletions. This powerful technology has not been available for nairoviruses, a deficiency that has seriously limited our understanding of CCHFV biology and pathogenesis. Here, we describe a method utilizing codon optimization of CCHFV’s L-RdRp to support the recovery of infectious CCHFV entirely from cDNA transfection. Using this system, we investigate the possible role of NSG cleavage in CCHFV life cycle.

## Results

### Recovery of recombinant CCHFV

We previously reported the reconstitution of transcriptionally active CCHFV RNPs by co-transfecting 2 plasmids producing the L-RdRp and N proteins together with a surrogate RNA minigenome synthesized by bacteriophage T7 RNA polymerase (T7 Pol) [9]. These minigenomes are composed of the viral promoter sequences (3′ and 5′ non-coding regions) of the S, M, or L, and a luciferase gene in place of the viral open reading frame (ORF). The co-transfection of a minigenome together with the L-RdRp and N proteins yielded luciferase activity [9], demonstrating the feasibility of reconstituting CCHFV RNPs. In order to recover infectious CCHFV from cloned cDNA, the complete S, M, and L segments were cloned between a T7 promoter to drive the transcription of CCHFV complementary genomic RNA copies, and a hepatitis D ribozyme to obtain authentic 3′ termini (S1A Fig).

Our first attempts consisted of transfecting 3 plasmids producing complementary (positive sense) copies of the CCHFV S, M, and L segments into cells stably expressing T7 (BSR-T7/5; S1 Fig). We chose to use positive-sense RNA segments over negative-sense, as uncapped T7 transcripts could be translated into enough viral proteins to launch CCHFV replication, as previously demonstrated for other bunyaviruses and arenaviruses [14–16]. In addition, most rescue systems for negative-strand RNA viruses rely on producing positive-sense transcripts to avoid the formation of double-stranded RNA between the negative-sense T7 transcripts and positive-sense support plasmids that are often required [17]. Transfection of only pT7-S, pT7-M, and pT7-L, failed to rescue CCHFV, possibly because the CCHFV L segment is extremely long compared to the L of arenaviruses and bunyaviruses and could make reconstituting RNPs less efficient.

**Fig 1:**
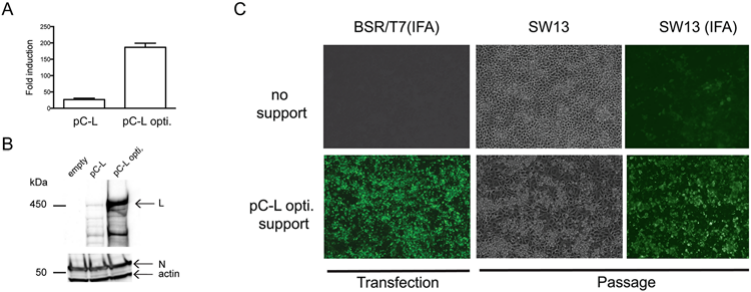
L-RdRp gene codon optimization and recovery of CCHFV from DNA. (A) Reporter minigenome luciferase activity was measured 48 h after transfecting BSR-T7/5 cells seeded in 10 cm^2^ wells. Cells were transfected with 250 ng of pC-L or pC-L opti, together with 500 ng of pC-N, 50 ng of pT7-M-Renilla [19], and 30 ng of internal control pGL3 per well. Data are represented as fold increase in *Renilla* luciferase expression over control transfections in which pC-L was omitted. Error bars indicate means ± standard deviation (n = 3) (B) V5-tagged L-RdRp and N protein levels in cell lysates from panel A as described before [9]. (C) BSR-T7/5 cells were transfected as presented in S1C Fig (pC-L support, upper panel), and also with pC-L opti in place of WT pC-L (lower panel). Four days post transfection, BSR-T7/5 cell supernatants were passaged onto SW13 cells, and viral antigens were detected with a GP38 domain-specific mAb (BSR-T7/5 IFA). Three days after passaging, viral cytopathic effect and CCHFV were visualized by bright field (SW13) or immunofluorescence (SW13 IFA) microscopy with anti-CCHFV hyperimmune mouse ascetic fluid (HMAF).

To improve reconstitution of RNPs containing full-length L, we supplemented the transfection reactions with expression vectors pC-L and pC-N (S1B Fig, pC-L + pC-N), producing the L-RdRp and N from a strong RNA polymerase II promoter that has been previously used to successfully replicate minigenomes of all CCHFV segments [9]. Despite multiple attempts and protocol modifications, no CCHFV was recovered using this set of plasmids. Western blot analysis of the L-protein from pC-L transfections revealed that only a small amount of the full-length L-RdRp had been produced [9]. We hypothesized that low abundance of full-length L-RdRp might be preventing the replication of full-length CCHFV RNA transcripts. Since bunyavirus transcription naturally occurs in the cytoplasm, we suspected that the L transcripts produced in the nucleus might yield truncated L-RdRp products due to aberrant splicing and/or premature termination of transcription by PolII. In an attempt to increase the amount of ~450 kDa L-RdRp product and improve viral RNA synthesis, we produced a synthetic V5-tagged L-RdRp codon optimized for expression in human cells (pC-L opti). The algorithms used to generate this L sequence maximize the usage of preferred human codons and remove potential cryptic splicing sites and secondary structures [18] that might interfere with transcription and translation of full-length L-RdRp. We first tested the effects of L gene codon optimization on L-RdRp activity using a luciferase minigenome system. Codon optimization led to ~7-fold increase in L-RdRp activity and more robust production of the full-length L-protein, confirming that codon optimization greatly improves L-RdRp activity and expression (Fig 1A and 1B). Therefore, we used pC-L opti instead of wild-type (WT) pC-L in subsequent attempts to rescue CCHFV.

**Fig 2:**
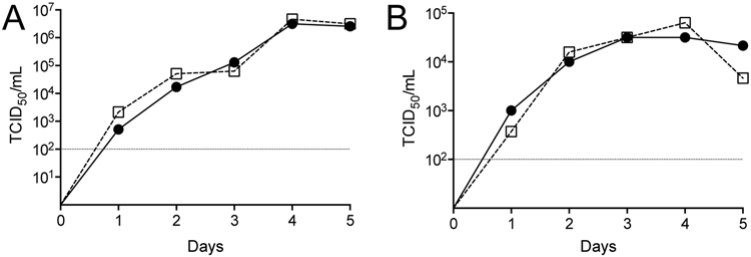
Growth kinetics of CCHFV derived from cDNA. (A) BSR-T7/5 and (B) A549 cells were infected with 0.001 of 50% tissue culture infective dose (TCID_50_)/cell of cDNA-derived CCHFV (circles) or parental virus isolate from Nigeria (squares). Viral titers were measured daily. Dashed line indicates the limit of detection.

Since CCHFV does not cause obvious cytopathic effect in permissive BSR-T7/5 cells, a PreGn mAb (7F5) was used to rapidly detect possible rescue of CCHFV directly in the transfected cells. This was possible because foci of > 10 cells with discernable Golgi-like perinuclear staining could only be observed when CCHFV was present as opposed to transfection of pT7-S,-M, and-L alone. Four days after transfection of BSR-T7/5 cells with pC-L opti, immunoreactive foci of infection were detected, suggesting the recovery of CCHFV from cDNA (Fig 1C). Supernatants from transfected BSR-T7/5 cells were then transferred to highly susceptible SW13 cells, which show apoptosis-induced cytopathic effect upon CCHFV infection [20]. Three days later, cytopathic effect were evident in these cells, and CCHFV antigens were detected throughout the cell monolayer. Growth kinetics of recombinant CCHFV in RIG-I signaling deficient (BSR-T7/5) [21] and competent (A549) cells matched those of the parental virus isolate (Fig 2). These data confirmed that infectious nairoviruses can be obtained entirely from cloned DNA in our system.

### Rescue optimization

**Fig 3:**
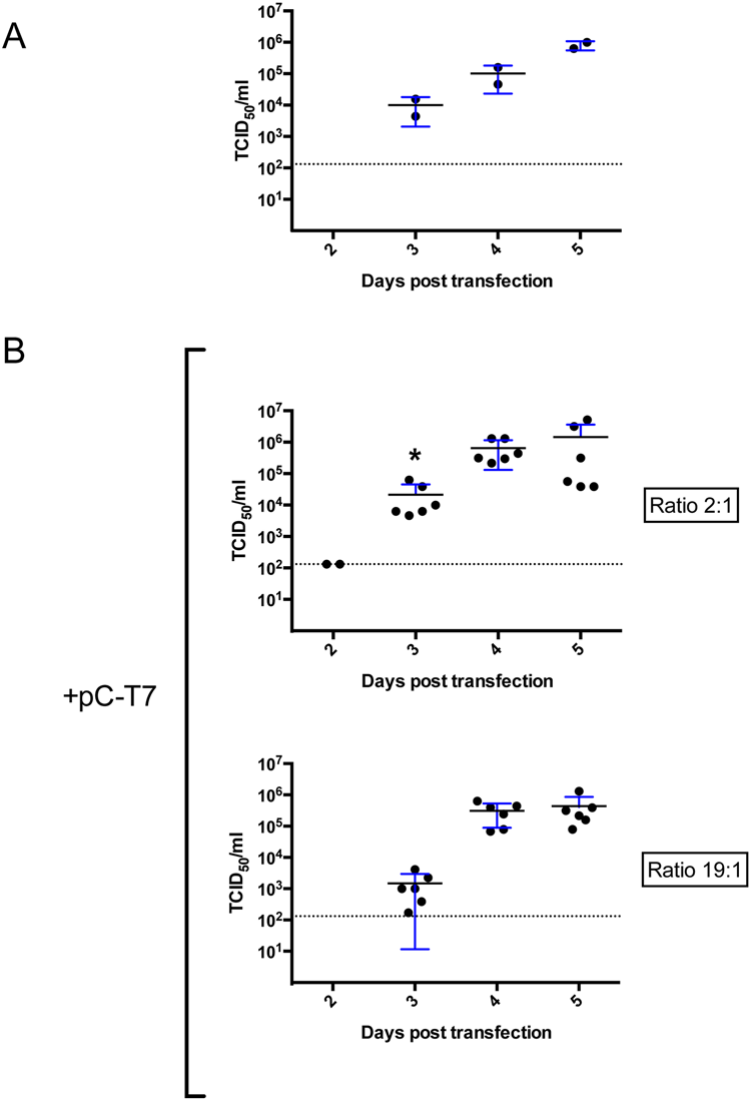
Optimization of support plasmid ratios for CCHFV rescue in BSR-T7/5. (A) BSR-T7/5 cells were transfected with 1 μg pT7-S, 2.5 μg pT7-M, 1 μg pT7-L, 0.66 μg pC-N, and 0.33 μg pC-L opti. Cell supernatants were collected and viral titers measured by determining TCID_50_ at the indicated times post transfection. (B) In the experiments using 2:1 ratio of pC-N to pC-L opti, cells were transfected as in panel A except that 1 μg of pC-T7 was added to the transfection mix. In the experiment using a 19:1 pC-N:pC-L opti ratio, the same plasmid mix was used as for the 2:1 ratio, but with 0.95 μg of pC-N and 0.05 μg of pC-L opti. Error bars indicate means ± standard deviation. Statistical significance was evaluated using Student’s unpaired *t* test. Asterisk (*) indicates P < 0.05 at 3 days post transfection (2:1 versus 19:1). Dashed line indicates the limit of detection.

After confirming CCHFV rescue, we optimized the system by varying the ratios of plasmids used in order to maximize rescue efficiency. We first tested the effect of adding exogenous T7 Pol. Without expressing plasmid-derived T7, we rescued CCHFV in 2 out 3 experiments (Fig 3A). In comparison, adding pC-T7 plasmid to the plasmid mix yielded rescue in 6/6 experiments and similar CCHFV titers over the 5-day experimental period (Fig 3B, ratio 2:1). We concluded that additional T7 is not required but overall not detrimental to rescue efficiency. Stable expression of T7 in BSR-T7/5 can vary with passaging and requires a strict antibiotic selection regimen [14], so being able to use exogenous T7 Pol can be useful.

Since adding pC-T7 was not detrimental to rescue efficiency, we continued to add pC-T7 in subsequent rescue experiments to mitigate any potential variation in the stable expression of T7 Pol from BSR-T7/5 cells. The ability to add T7 also allowed us to determine if CCHFV could be efficiently rescued in cell lines that do not stably express T7. The human hepatocyte cell line Huh7 was transfected with pC-T7, pC-N, pC-L opti and pT7-S,-M, and-L; virus was detected, and viral titers were measured by tissue culture infective dose (TCID_50_) determination 2–5 days post transfection. CCHFV was rescued from Huh7 cells in 3/3 experiments, and infectious virus titers peaked at 4 days post transfection (S2 Fig) with no significant differences from levels seen in BSR-T7/5 cells when using a 2:1 pC-N:pC-L opti plasmids ratio (Fig 3B, ratio 2:1).

We next determined if increasing support plasmid ratios affected rescue efficiency. Recombinant CCHFV was detected as early as 2 days post transfection when pC-N and pC-L opti were used in a 2:1 ratio, and ~10-fold higher viral titers were obtained 3 days post transfection than when using a 19:1 ratio of these plasmids (Fig 3B). However, rescue efficiency remained 100% with both ratios tested, and viral titers peaked 4–5 days post transfection independently of the ratios of support plasmids used.

**Fig 4:**
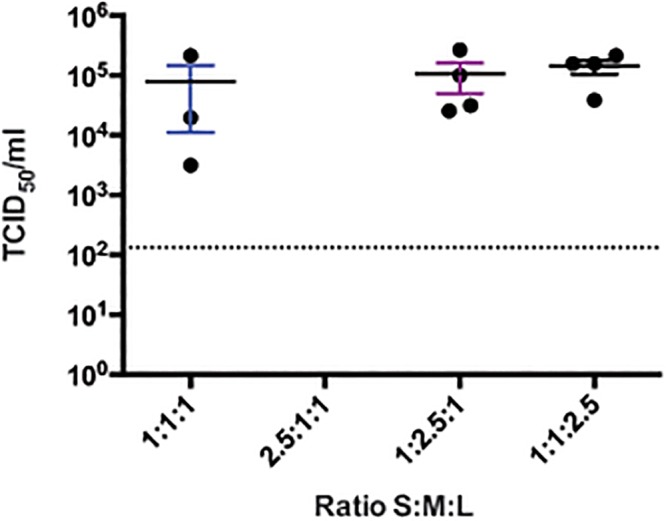
CCHFV rescue efficiency using varying ratios of plasmids producing complementary genome segments in BSR-T7/5. BSR-T7/5 cells were transfected with a total of 4.5 μg of pT7-S, pT7-M, and pT7-L at the indicated S:M:L ratios, together with 0.66 μg pC-N, 0.33 μg pC-L opti, and 1 μg pC-T7. Supernatants were collected 4 days after transfection, and viral titers were measured by TCID_50_ determination. Dashed line indicates the limit of detection.

Finally, we varied the ratios of plasmids used to express the full-length complement RNA of the 3 CCHFV segments (pT7-S, pT7-M, and pT7-L) while keeping the 2:1 ratio of support plasmids (Fig 4). At 4 days post infection, the same viral titers of ~1 × 10^5^ TCID_50_/mL were obtained at all ratios tested, except for the 2.5:1:1 (S:M:L) ratio, which did not yield measurable CCHFV titers.

### Role of furin in CCHFV infection

**Fig 5:**
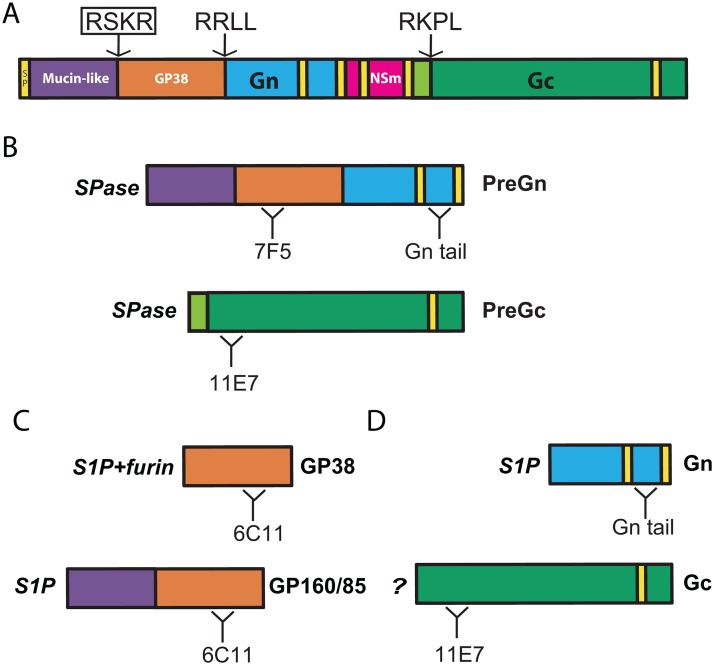
CCHFV M segment polyprotein processing. (A) Signal peptide (SP) and predicted transmembrane domains are indicated in yellow. Arrows indicate locations of cleavage motifs recognized by mammalian convertases. The RSKR motif (boxed) was mutated to ASKA to block processing. (B) Signal peptidase (SPase) cleavages result in production of Gn and Gc precursors (PreGn and PreGc). Binding regions of the anti-glycoprotein antibodies used in this study (7F5, Gn tail, 11E7, and 6C11) are also represented. (C) Mature glycoprotein products (GP160/85, GP38, Gn, and Gc) resulting from mammalian convertase cleavage. (D) S1P cleavage is required for the production of GP160/85 and Gn, while GP38 requires cleavage by both S1P and furin-like PCs. An unidentified mammalian convertase is required for PreGc maturation to Gc.

Within the family *Bunyaviridae*, only nairoviruses produce NSGs or viral glycoprotein precursors subjected to PC cleavage [8,10,22]. In most cases, the cleavage of structural viral glycoproteins promotes viral replication by allowing the entry of the virus into host cells. Interestingly, Ebola virus, a filovirus that causes highly fatal hemorrhagic fever, synthesizes NSGs and a structural glycoprotein precursor containing MLD and furin-like PC cleavage motifs (RTRR↓) [23,24]. Unlike most viral glycoproteins cleaved by furin-like PCs, Ebola virus glycoprotein cleavage appeared to have little or no effect on viral replication, as infectious virus was still produced in mutants with blocked processing of the glycoprotein precursor [25,26]. We therefore endeavored to determine whether cleavage of CCHFV glycoprotein at the RSKR_247_ site was critical for CCHFV propagation (Fig 5).

**Fig 6:**
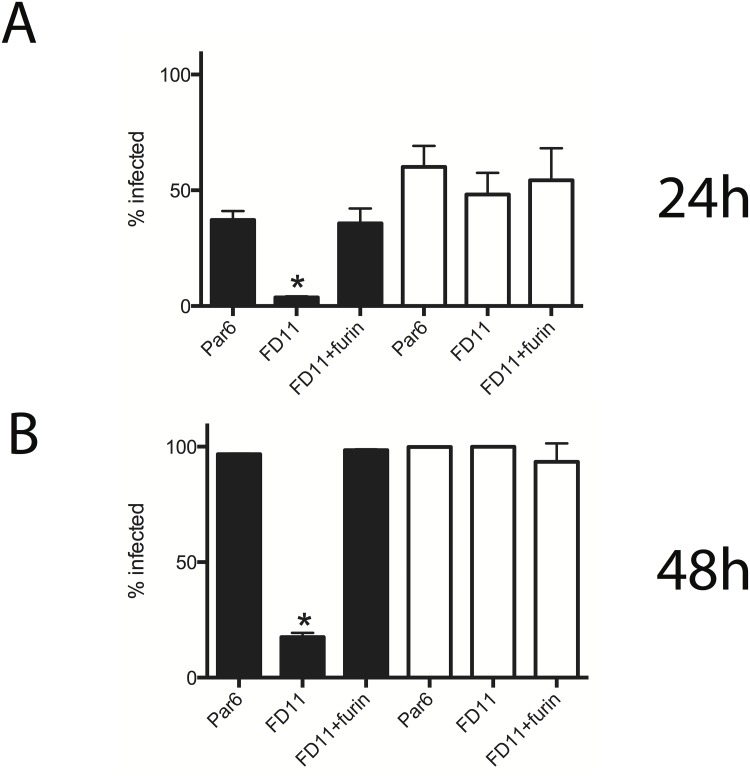
Furin enhances CCHFV propagation. CHO-derived cell lines used were parental clone 6 (Par6), furin-deficient (FD11), and FD11 stably expressing furin (FD11-Fur). Each cell line was infected with CCHFV at multiplicity of infection (MOI) = 1 or with Rift Valley fever virus expressing EGFP in place of NSs (RVFV-EGFP; MOI = 0.1). Percentage of infected cells was determined by immunostaining for CCHFV, or by EGFP detection for RVFV at 24 h (A) and 48 h (B) post infection. Black bars represent CCHFV-infected cells; white bars represent RVFV-infected cells. Error bars indicate means ± standard deviation (n = 3). Statistical significance was evaluated using Student’s unpaired *t* test. Asterisk (*) indicates P < 0.01.

The importance of furin-like PCs in the propagation of CCHFV and control Rift Valley fever virus (RVFV) was evaluated by infecting cells deficient in furin. The parental Chinese hamster ovary (CHO)-derived cell lines (Par6), furin-deficient (FD11) [27], and furin-reconstituted FD11 (FD11-Fur) were infected with CCHFV or RVFV at multiplicity of infection (MOI) of 1 or 0.1, respectively, and percentages of infected cells were determined 24 and 48 h post infection (Fig 6A). After 24 h, 37% of Par6 cells and 36% of FD11-Fur cells were infected with CCHFV, compared to 4% of FD11 cells not expressing furin. After 48 h, Par6 and FD11-furin cell monolayers were totally infected, while only 18% of the FD11 cells where infected (Fig 6B). In comparison, the replication of RVFV, which does not contain known furin-like PC cleavage motifs in its glycoprotein sequence, was similar in all cell lines, supporting the hypothesis that glycoprotein cleavage at a putative RSKR motif enhances CCHFV replication. Alternatively, furin might indirectly regulate CCHFV spread by processing host proteins implicated in its life cycle.

### Generating CCHFV producing endoproteolysis-resistant NSGs

To clarify whether furin or general NSG cleavage is required for CCHFV replication, we first sought to recover a CCHFV with a glycoprotein resistant to furin-like PC endoproteolysis. The substrates of furin-like PCs are normally cleaved at the general (R/K)-2nX-R↓ motif, where n = 0–3 amino acids [28]. The canonical motif (RSKR↓) is located at the junction of the MLD and GP38 domains of CCHFV (Fig 5A). To block processing at this site, we mutated the ORF encoded by the pT7-M plasmid, so that Arg residues at positions 1 and 4 were changed to Ala (RSKR to ASKA). Given the specificity of furin-like PCs, these mutations should block the conversion and production of GP38. The pT7-M-ASKA plasmid was transfected together with plasmids needed to produce recombinant CCHFV, and a viable recombinant virus (CCHFV-ASKA) was recovered. Like WT CCHFV, the rescued CCHFV-ASKA mutant produced extensive cytopathic effect in SW13 cells. In cells infected with CCHFV-ASKA, PreGn colocalized with a Golgi marker, similarly to WT CCHFV (S3 Fig). Sequencing the M segment from this mutant virus confirmed mutations only at the cleavage site mutations.

**Fig 7:**
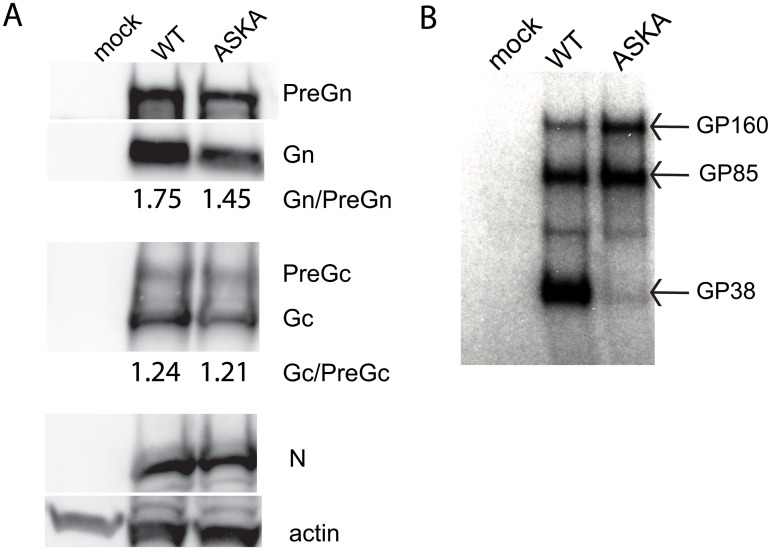
Effects of blocking furin cleavage on CCHFV glycoprotein maturation. (A) SW13 cells were infected with WT CCHFV or CCHFV-ASKA at MOI = 0.1, and immunoblots of structural proteins were performed on lysates collected 24 h post infection. Ratios of Gn:Pre and Gc:PreGc were obtained by densitometry of the bands (AlphaView; Alpha Innotech). (B) Immunoprecipitation of secreted non-structural proteins containing the GP38 domain with 6C11 mAb.

To confirm that the ASKA mutation blocked NSG processing, viral proteins extracted from lysates of infected cells were analyzed using antibodies directed against PreGn/Gn, PreGc/Gc, and N. Densitometry analysis of the precursor and mature Gn and Gc indicated no change in Gc:PreGc ratios, while the Gn:PreGn ratio was slightly reduced in ASKA mutants (Fig 7A). The anti-GP38 antibody also detected PreGn but GP160/85 and GP38 did not accumulate in cells to detectable levels (S4 Fig).

To detect the secreted NSGs, supernatants of infected cells were immunoprecipitated using a mAb directed against the GP38 domain. Polyacrylamide gel electrophoresis revealed GP160/GP85 and GP38 presence upon infection with WT CCHFV (Fig 7B). In comparison, CCHFV-ASKA infection produced abundant GP160/GP85, but only trace amounts of GP38. Thus, mutating RSKR to ASKA selectively impaired maturation of the glycoprotein precursor to GP38, and slightly reduced PreGn conversion to Gn.

### Direct role of furin cleavage at RSKR motif in virion production

**Fig 8:**
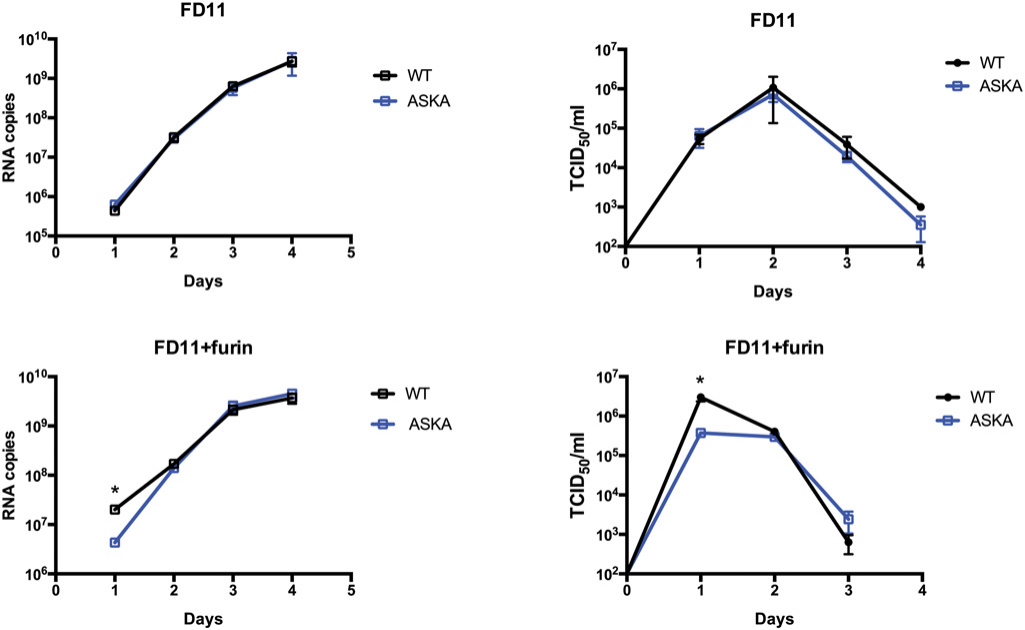
Furin effect on CCHFV-WT and-ASKA growth. FD11 and FD11-Fur cells were infected with CCHFV-WT or CCHFV-ASKA (MOI = 0.1). Cell supernatants were collected daily, and RNA S-segment copy numbers and infectious virus titers were measured by qRT-PCR and TCID_50_ determination, respectively. Means ± standard deviation (n = 3) are plotted. Statistical significance was evaluated using Student’s unpaired *t* test. Asterisk (*) indicates P < 0.05.

Since CCHFV-ASKA mutations effectively blocked the production of GP38, we addressed the role of GP38 in the production of infectious CCHFV. FD11 and FD11-Fur cells were infected with CCHFV-WT or CCHFV-ASKA, and viral loads and associated viral RNA levels were measured for 5 days after infection by, respectively, TCID_50_ determination and qRT-PCR (Fig 8). Furin-reconstituted cells (FD11-Fur) infected with CCHFV-WT yielded ~10-fold more infectious virus and viral RNA than the same cells infected with CCHFV-ASKA, but only 1 day post infection. In comparison, no differences were noted in furin-deficient cells (FD11; Fig 8).

## Discussion

Despite increasing numbers of reported CCHF cases and disease severity, the life cycle of CCHFV remains poorly defined. Here, we present an efficient method to recover recombinant infectious CCHFV entirely from cDNA. This technical accomplishment should facilitate investigations of CCHFV replication cycle, pathogenesis, and transmission. Furthermore, we demonstrate the utility of this reverse genetics virus rescue system by generating a specifically designed CCHFV mutant, and provide direct evidence that cleavage at RSKR by furin-like PCs enhances the virion production of this severe pathogen.

### Rescue system development and optimization

Prior to codon optimization of the L-gene in the support plasmid, all attempts to generate CCHFV failed. Notably, the same plasmid vector stocks expressing complementary copies of the 3 viral segments (pT7-S, pT7-M, and pT7-L) were used in all of our unsuccessful and successful attempts, suggesting that inefficient synthesis of full-length L-RdRp might explain our initial inability to rescue recombinant CCHFV (Fig 1). The increase in L-RdRp expression upon introducing the optimized L construct was seen in both L-RdRp yield and activity (Fig 1A). The recoded, optimized L (pC-L opti) efficiently launched viral replication and allowed us to obtain infectious CCHFV with a tailored genome. CCHFV infection persists in ticks and its replication cycle is exclusively cytoplasmic. It is plausible that evolutionary pressure exerted by the tick host cells and adaption for cytoplasmic transcription could explain the difficulty in producing large amounts of L-RdRp from the nucleus of mammalian cells using CCHFV’s natural codons (Fig 1B). Our failure to recover CCHFV when using WT pC-L in previous attempts suggested that *in silico* recoding may be important for systematically recovering recombinant CCHFV.

Synthetic genes can also be introduced into the genome of viruses using reverse genetics. Introduction of rare codon pairs in poliovirus and influenza virus genes leads to reduced viral protein production, strong attenuation *in vivo*, and prototype vaccines that can confer complete protection against homologous virus challenges [29,30]. As efficacious CCHF vaccines are critically needed, a similar codon deoptimization approach could now be attempted in order to obtain a live, attenuated CCHFV vaccine candidate.

Robust recue systems are critical for obtaining mutant viruses, especially those with severe growth defects. Herein, we describe optimization of the rescue protocol that yielded recombinant CCHFV in virtually every transfection attempted. Although not required for rescue in cells stably expressing T7 Pol, exogenously added pC-T7 did not negatively impact rescue efficiency in BSR-T7/5 cells. Adding pC-T7 further allowed us to rescue CCHFV in Huh7 cells, a human hepatocyte cell line not stably expressing T7 Pol. This demonstrates that our rescue system is robust and could be easily transposed to a cell line of human origin without cell specific optimization. The option of using other cell lines might be particularly important in the eventuality of mutations preventing CCHFV recovery in BSR-T7/5 due to low or absent expression of cell- or species-specific host factors.

Optimal rescue conditions should yield the highest viral titers at early times post-transfection. By varying support plasmid ratios (pC-N:pC-L opti), we observed that lower ratios increased CCHFV yield by ~10-fold 3 days post transfection, showing that reducing the expression of codon-optimized L-RdRp is detrimental to virus rescue. Nevertheless, even lower amounts of codon-optimized L-RdRp eventually yielded high titers of CCHFV at later time points. In contrast, increasing the ratio of pT7-S to pT7-M and-L dramatically reduced virus rescue efficiency, as noted by the absence of detectable CCHFV at 4 days post transfection when using an S:M:L ratio of 2.5:1:1 (Fig 4). This finding suggests that overrepresentation of the plasmid producing the S segment complementary genome should be avoided. The smaller size of pT7-S could explain these phenomena, as T7 Pol. may preferentially synthesize smaller segments more efficiently, thereby requiring less pT7-S input than pT7-M and pT7-L.

### The role of furin-like cleavage in efficient production of CCHFV

Limited endoproteolysis is a common mechanism exploited by viruses to modulate the activity of their glycoprotein precursors, and CCHFV uses PCs with non-overlapping specificity to generate GP38 and Gn. Previous biochemical analyses of the M-polyprotein have revealed the role of host proteases in the complex biosynthesis of at least 8 different proteins from a single protein [6,8]. Our past efforts highlighted the critical function of S1P in assembling infectious CCHFV [11], but the contribution of furin-like PCs in viral replication remained unexplored. Using the CCHFV reverse genetics system, we addressed the function of this cleavage on CCHFV replication without the need to rely on furin-like PC inhibitors. This is particularly important, as inhibition of furin-like PCs could also modulate the activity of other cellular receptors, ligands, growth factors, and enzymes that could indirectly affect CCHFV replication independently of glycoprotein processing. The conservation of basic amino acids in the RSKR motif among all CCHFV strains from diverse endemic areas (S5 Fig) suggests selective pressure for furin-like PC cleavage at this particular site.

Structural analysis of RVFV Gc revealed that the overall architecture of bunyavirus Gc resembles that of class II fusion proteins of West Nile virus and Sindbis virus, whose companion proteins are cleaved by furin-like PCs. However, neither RVFV Gc nor the companion protein Gn is obtained by PC activity. Therefore, it became critical to determine whether cleavage of CCHFV glycoprotein, both by furin-like PCs at the putative RSKR motif and by SKI-I/S1P at the RRLL motif, is necessary for efficient spread and production of CCHFV, or whether furin-like PC cleavage simply fulfills accessory functions dispensable for viral replication. To address the function of CCHFV glycoprotein cleavage at the RSKR motif in virus propagation, we obtained a recombinant CCHFV variant, CCHFV-ASKA, which produced glycoprotein precursors resistant to furin-like PC activity. The simple recovery of ASKA mutant virus demonstrated that furin-like PC cleavage is not an essential step in CCHFV replication in cell culture, and enabled us to study the function of cleavage at this site without being obligated to manipulate cellular functions with inhibitors, siRNA, or gene overexpression.

Consistent with the preference of furin-like PCs for Arg residues at P1 and P4, the CCHFV-ASKA glycoprotein profile confirmed that mutating P1 and P4 selectively blocked glycoprotein processing to GP38. To a less significant extend, mutations reduced the Gn:PreGn ratio, but the Gc:PreGc ratio was unaffected. The fact that mature Gn was still produced is compatible with the rapid production of PreGn by S1P early in the secretory pathway (endoplasmic reticulum/early Golgi); furin, on the other hand, is localized in the trans-Golgi network, and the cleavage yielding GP38. Consequently, we propose that S1P PreGn cleavage into Gn and MLD-containing NSGs (GP160/85) precedes furin-like PC cleavage, and that the distinct localization of S1P and furin might dictate the proper sequence of cleavage events that yields the complete set of structural and non-structural glycoproteins. However, our data also suggest that furin processing could indirectly increase PreGn to Gn conversion performed by S1P. It remains to be shown if furin does indeed cleave a pool of PreGn prior to S1P and if prior cleavage at RSKR site would enhance Gn production.

The relative ease of propagating the CCHFV-ASKA mutant appears in stark contrast with the total loss of CCHFV infectivity when S1P is not expressed [11]. Further experiments were performed to address the function of furin-like PCs in the replication of CCHFV, using RVFV as a PC-independent control. Although our data do not exclude the potential role of other furin substrates in CCHFV’s life cycle, our findings certainly stress a specific role of furin cleavage at the RSKR site. This conclusion is supported by higher WT CCHFV production than CCHFV-ASKA production in cells expressing furin (Fig 8). If furin cleavage at RSKR did not contribute to CCHFV’s replication, then this difference would also be expected in furin-deficient cells. Therefore, we conclude that cleavage at the putative furin-like PC motif is not essential for CCHFV production, but furin cleavage at RSKR enhances virion production.

Despite evolutionary conservation of the RSKR motif, our *in vitro* replication experiments confirm that this cleavage event is dispensable for the production or cell-to-cell spread of CCHFV. The biological relevance of CCHFV NSGs remains unknown, and no specific ligands have been determined for any of them. Intriguingly, the decrease in CCHFV-ASKA production was transient, and CCHFV-ASKA levels matched those of WT-CCHFV 2 days and later post infection, perhaps because furin cleavage affects virion production early during the infection when the levels of glycoproteins are lower. Studies focused on NSG function in conjunction with detailed biosynthesis of the various M-polyprotein glycoprotein products are warranted to precise the function of furin cleavage of CCHFV glycoproteins. Even though modest, reduction in PreGn to Gn certainly merits further consideration given the essential role of S1P in Gn biosynthesis [10] and CCHFV infectivity [11]. But how could furin cleavage at RSKR site increase S1P cleavage at RRLL? One possibility could be trimming of PreGn from its MLD by furin. In this scenario, PreGn would escape S1P cleavage and travel to the trans-Golgi network, where furin would trim off its MLD (Fig 5); this, in turn, would facilitate the kinetics of Gn production and subsequent virion production. CCHFV assembly occurs at the Golgi or trans-Golgi network, and after assembly, the intracellular vesicles containing viruses are transported toward the cell surface, where they are released by exocytosis. CCHFV GP38 is secreted by exocytosis, but is not tightly associated with extracellular CCHFV particles [8]. Since we observe a reduction in CCHFV-ASKA production in the extracellular milieu and GP38, we speculate that GP38 could be involved in the efficient sorting and/or release of mature CCHFV particles. We propose that future studies should consider the possible impact of furin cleavage of the MLD on Gn formation and investigate the role of GP38 on CCHFV production.

Recently, an experimental *in vivo* model of CCHFV transmission from ticks to vertebrates [31], and a model of CCHF pathogenesis [32] in mice, were reported. These *in vivo* systems, combined with the power of reverse genetics, offer unique opportunities to characterize CCHFV protein functions in various aspects of virus pathogenesis and biology. Our study lays important groundwork to further define how NSGs and their cleavage by furin-like PCs affect CCHFV biology and pathogenesis in the mouse model, and the persistence and transmission of CCHFV in the tick vector. The function of NSGs is particularly intriguing because filoviruses from the genus *Ebolavirus* also secrete glycoproteins that contain an MLD and are cleaved by furin-like PCs. Therefore, it is critical to better characterize the function of these secreted glycoproteins that might share a common mechanism to cause severe hemorrhage and death in humans.

In conclusion, this work highlights the ability of experimentally producing CCHFV variants entirely from cDNA. Using CCHFV reverse genetics, we efficiently recovered WT virus and a virus variant encoding a glycoprotein precursor resistant to furin-like PC cleavage. This mutant CCHFV was used to specifically examine the role of furin-like PC cleavage on virus propagation. In the future, this reverse genetics systems could be used to experimentally identify genetic determinants of virulence, critical functions of viral proteins, non-coding regions of the viral genome, and in the rational design of live attenuated vaccines.

## Materials and Methods

### Cells and viruses

SW13 cells, a generous gift from P. Leyssen (Rega Instituut KU Leuven, Belgium), were cultured in DMEM supplemented with 10% FBS and 1 mM sodium pyruvate. BSR-T7/5 were a generous gift from K.K. Conzelmann (Ludwig-Maximilians-Universität, Munich, Germany), and maintained as described before [14]. Vero-E6 and A549 (both from American Type Cell Collection) cells were maintained in DMEM + 10% FBS. All cell culture media were supplemented with 100 units/mL of penicillin and streptomycin. Parental CHO-derived cells (Par6), furin-deficient Par6 cells (FD11), and FD11 stably expressing furin (FD11-Fur) were kindly provided by Stephen Leppla (National Institute of Health, Bethesda, MD, USA); these cell lines were maintained in MEM-alpha medium with 10% FBS. CCHFV strain IbAr10200 (former Yale Arbovirus Research Unit, CT, USA) was isolated in Nigeria in 1965 from ticks, and has a passaging history in suckling mice and Vero-E6 cells.

### Plasmids

CCHFV RNA was extracted with Tripure reagent (Roche Diagnostics Corp, Indianapolis, IN, USA) using manufacturer’s protocol. Full-length cDNA from the S, M, and L segments was obtained by reverse transcription of CCHFV RNA at 50°C (Thermoscript RT, Invitrogen, Grand Island, NY, USA) using a DNA primer complementary to the 3′ end of the viral genomic RNAs. Complete cDNA were sequence-amplified by PCR using high fidelity Phusion enzyme (New England Biolabs, Ipswich, MA, USA) and cloned into the pT7 vector. The pT7 vector was previously described as V (0.0)/B [14], and contains a BsmB1 cloning site located between a T7 promoter and a hepatitis D ribozyme T7 polymerase terminator motif [14]. The S, M, and L cDNAs were cloned into the BsmB1-digested pT7 vector in viral complementary orientation relative to the T7 promoter (S1 Fig). Sequences of all plasmids used to rescue CCHFV are available in GenBank. Primary T7 transcripts derived from pT7-S (KJ648914), pT7-M (KJ648915), and pT7-L (KJ648913) contain an artificial G at the 5′ ends, but this nucleotide is rapidly lost upon replication [9]. pC-N (KJ648912), pC-L (KJ648911), and T7-M-Renilla constructs were described previously [9,19]. pT7-M-ASKA (KJ648916) was obtained by PCR mutagenesis of pT7-M using QuickChange Lightning Site Directed Mutagenesis Kit (Agilent Technologies, Santa Clara, CA, USA) per manufacturer’s instructions. pC-L opti (KJ648910) was obtained by optimizing the codons of CCHFV IbAr10200 L ORF for maximal expression in human cells [18] (GeneArt, Ratisbonne, Germany). Both pC-L and pC-L opti were tagged with an N-terminal V5 epitope. pC-T7 [33] was obtained by cloning human codon-optimized bacteriophage T7 RNA Pol (Genscript, Piscataway, NJ, USA) into pCAGGS.

### Minigenome and virus rescue

The T7-M-Renilla minigenome has been previously described [19]. This minigenome is based on our previous *Gaussia* luciferase version that necessitated transfection of *in vitro* minigenome transcripts to minimize background activity [9]. In combination with codon optimization of the L-polymerase, changing *Gaussia* to *Renilla* luciferase reporter yielded negligible background levels, thereby eliminating the requirement of producing *in vitro* minigenome transcripts. All attempts to recover CCHFV were performed in BSL-4 facilities at the Centers for Disease Control and Prevention (Atlanta, GA, USA). Six-well plates were seeded with 3.5 × 10^5^ BSR-T7/5 cells/well 1 day prior to transfection in 3 mL of DMEM supplemented with 5% FBS. 16–24 h later, cells were transfected with the indicated amounts of pT7-S, pT7-M, pT7-L, pC-L opti, pC-N, and optionally with pC-T7, combined with 2.5 μL of Mirus LT1 transfection reagent (Mirus Bio, Madison, WI, USA) per μg of DNA in 250 μL of OPTI-MEM (Life Technologies, Grand Island, NY, USA). For CCHFV-ASKA rescue, pT7-M was substituted with pT7-M-ASKA. 16–24 h post transfection, 5 mL of DMEM with 2% FBS was added to each transfected well. Cell supernatants were harvested 4 days post infection, and 2 mL of supernatant were passaged to a 75 cm^2^ confluent flask of SW13 cells grown in DMEM with 10% FBS and 1 mM sodium pyruvate. All viruses were titrated using a standard TCID_50_ protocol in SW13 cells.

### Immunofluorescence

Cells were fixed with 10% formalin buffered solution. BSR-T7/5 cells were incubated with a CCHFV-specific glycoprotein antibody (7F5, USAMRIID, Fort Detrick, MD, USA) or with hyperimmune mouse ascetic fluid (HMAF) for SW13 cells, followed by incubation with goat anti-mouse Alexa 488-conjugated antibody (Life Technologies). To determine subcellular localization of CCHFV glycoprotein, Vero-E6 cells were infected with CCHFV-WT or CCHFV-ASKA, and stained with 7F5 mAb and rabbit anti-giantin pAb (Covance, Princeton, NJ, USA), followed by incubation with DAPI to stain nuclei, goat anti-mouse Alexa 488-conjugated secondary antibody, and goat anti-rabbit Alexa 546-conjugated secondary antibody. Images were captured with a TCS SP5 confocal microscope (Leica Microsystems, Buffalo Grove, IL, USA).

### Western blotting and metabolic labeling

Western blotting and metabolic labeling were performed as previously described [11,12]. Briefly, Gn, Gc and glycoprotein precursors were detected with rabbit anti-PreGn/Gn (kindly provided by A. Mirazimi, Folkhälsomyndigheten, Sweden) and anti-PreGc/Gc mAb 11E7 (USAMRIID), while N was detected with CCHFV HMAF. For metabolic labeling, SW13 cells were infected with CCHFV at MOI = 0.1 for 6 h. Cells were pulsed overnight with 100 μCi/mL of S_35_-labeled Met and Cys in normal DMEM supplemented with 1% of low-IgG FBS (Life Technologies). Cell supernatants were cleared of contaminating bovine IgG using protein A/G magnetic beads (Thermo Scientific, Grand Island, NY, USA) before immunoprecipitating secreted GP38-containing glycoproteins with 8C11 mAb, kindly provided by A. Garrison and C. Schmaljohn (USAMRIID). Immunoprecipitated proteins were separated on Tris-acetate NuPAGE 4–8% gradient gels, and labeled proteins were revealed by autoradiography.

### Analysis of CCHFV- and RVFV-infected cell cultures

CHO-derived cell lines Par6, FD11, and FD11-Fur, seeded in MEM-alpha medium with 5% FBS, were infected with recombinant CCHFV WT or RVFV-ΔNSs:GFP-ΔNSm [34], and fixed in 10% formalin buffered solution. CCHFV-infected cultures were incubated with CCHFV HMAF and goat anti-mouse Alexa 488-conjugated antibody. Nuclei and cells were respectively counterstained with Hoechst 33342 and HCS CellMask Red stain (Life Technologies). Images were acquired with a 20× objective on an Operetta High Content Imaging system (PerkinElmer Inc, Waltham, MA, USA). To detect RVFV-infected cells, EGFP was measured.

### Quantitative RT-PCR

RNA was isolated from cell supernatants using Magmax technology (Life Technologies) and subjected to one-step qRT-PCR using SuperScript III Platinum One-Step qRT-PCR Kit (Life Technologies). CCHFV S segment amplification was performed on an Applied Biosystems 7500 Real-Time PCR System using sense (5′ATGAACAGGTGGTTTGAAGAGTT 3′) and antisense (5′TGGCACTGGCCATCTGA 3′) primers, and TaqMan probe (5′[6-carboxy-fluorescein (FAM)] TGTCCAAATTGGGAACACTCTCGCA [BlackBerry Quencher (BBQ)] 3′) (TIB Molbiol, Adelphia, NJ, USA).

## Supporting Information

S1 Fig Design of plasmids and strategies to recover CCHFV from cDNA transfection. (A) Full-length complementary copies of the viral RNA segments were cloned between a T7 RNA promoter (T7, green box) and hepatitis D ribozyme (HDR, red box). The orientations of the viral large (L; blue), medium (M; green), and small (S; red) segment open reading frames (ORFs) are indicated by arrows. (B) Support plasmids pC-L and pC-N, designed to produce the L RNA-dependent RNA polymerase (L-RdRp) and nucleoprotein, respectively, were generated by introducing the L (blue) and S (red) ORFs into the mammalian expression vector pCAGGS. Recombinant pC-L, or codon-optimized support L-RdRp (pC-L opti), and pC-N vectors contain an RNA polymerase II promoter (PolII, orange box) with a CMV early enhancer, a chicken ß-actin promoter, and a polyadenylation signal (polyA, black box). (C) The minus (-) and plus (+) support plasmid combinations used in our initial attempts to recover CCHFV from plasmid transfections. In the—support experiment, pT7-L, pT7-M, and pT7-S were transfected into BSR-T7/5 cells alone, and in the + support, pT7-L, pT7-M, and pT7-S were co-transfected with pC-N and pC-L or pC-L opti. BSR-T7/5 cells stably produce T7 RNA polymerase (T7, green oval) that transcribes genomic complementary copies from transfected pT7 plasmids. In a successful recovery attempt, the T7 transcripts would generate ribonucleoprotein particles represented by beads colored in green (nucleoprotein) and gray (L-RdRp). Ribonucleoprotein particles from the 3 RNA segments are incorporated into nascent virions to obtain infectious CCHFV.
(EPS)

S2 Fig CCHFV rescue efficiency in Huh7 cells. Huh7 cells were transfected with 1 μg pT7-S, 2.5 μg pT7-M, 1 μg pT7-L, 0.66 μg pC-N, 0.33 μg pC-L opti, and 1 μg of pC-T7. Cell supernatants were collected, and viral titers measured by TCID_50_ determination at indicated times post transfection. Dashed line indicates the limit of detection.
(EPS)

S3 Fig Localization of glycoprotein resistant to furin cleavage. Vero-E6 cells were infected with wild-type CCHFV (WT) or CCHFV-ASKA (ASKA) for 24 h. Subcellular localization of PreGn was compared to Golgi apparatus marker giantin (Golgi).
(TIFF)

S4 Fig GP38 is not detectable in lysates of infected cells. Par6 cells were infected with CCHFV-WT or CCHFV-ASKA at MOI of 0.1. Proteins from the cell lysates were separated by NuPAGE (Bis-Tris 4–12% gels in MOPS buffer). Membranes were blotted with anti-GP38(379–392) pAb [8]. Note that PreGn is detectable with GP38-specific antibody.
(EPS)

S5 Fig Alignment of glycoprotein precursor regions from various Crimean Congo hemorrhagic fever virus (CCHFV) strains. Complete sequences of CCHFV strains were aligned using CLC Main Work Bench 7.0. Arrow indicates site of furin-like proprotein convertase (PC) cleavage. The RSKR cleavage motif is completely conserved in all CCHFV strains.
(EPS)
