# Supplementary figures and images for: Recovery of Recombinant Crimean Congo Hemorrhagic Fever Virus Reveals a Function for Non-structural Glycoproteins Cleavage by Furin
Source: PLoS Pathog. 2015 May 1;11(5):e1004879. doi: 10.1371/journal.ppat.1004879 (PMC4416775; doi:10.1371/journal.ppat.1004879)

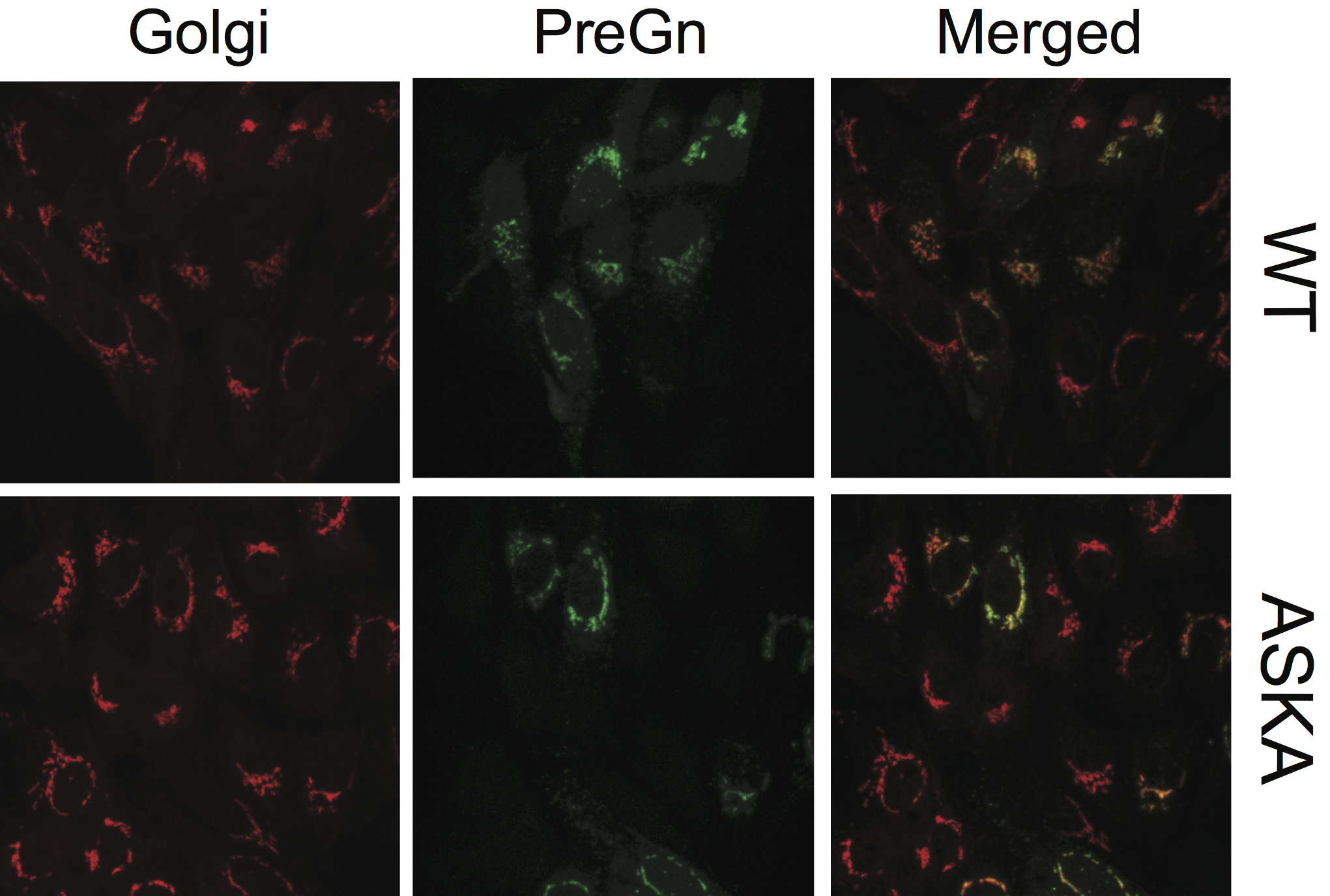

Supplement: S3 Fig — Vero-E6 cells were infected with wild-type CCHFV (WT) or CCHFV-ASKA (ASKA) for 24 h. Subcellular localization of PreGn was compared to Golgi apparatus marker giantin (Golgi). (TIFF) [file ppat.1004879.s003.tiff]
